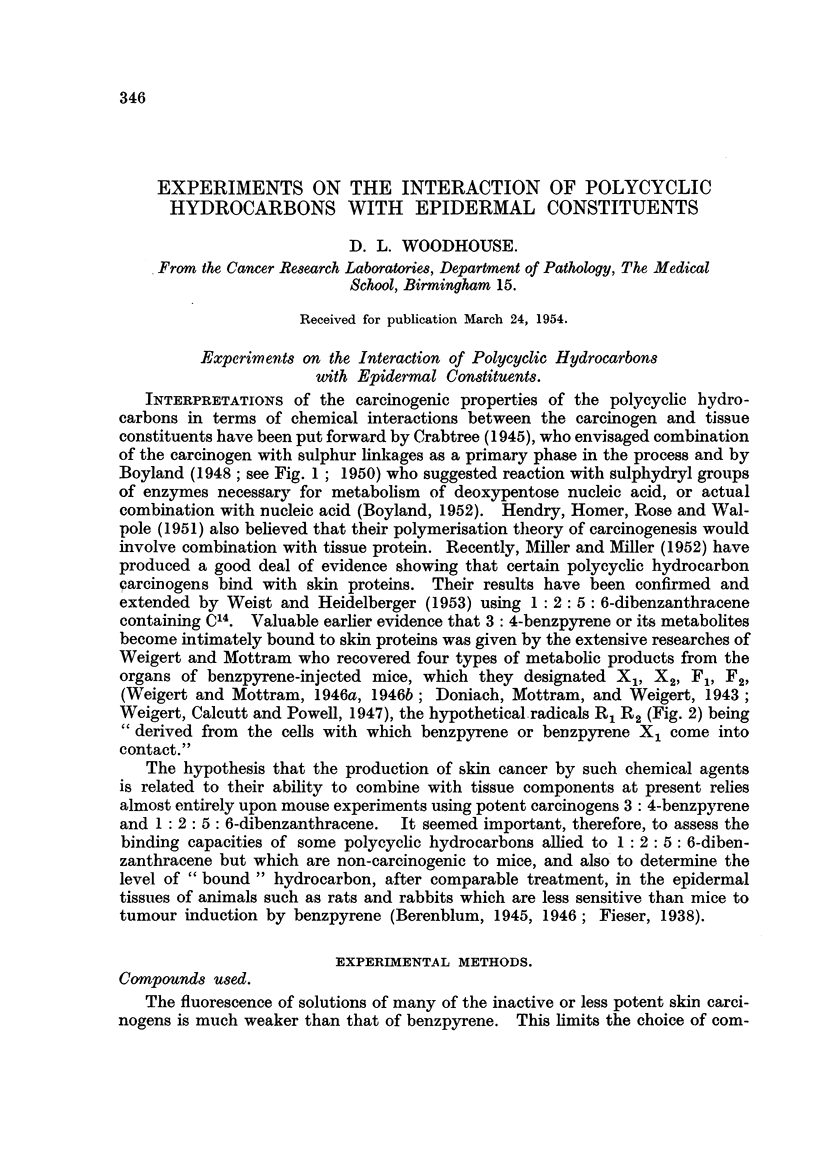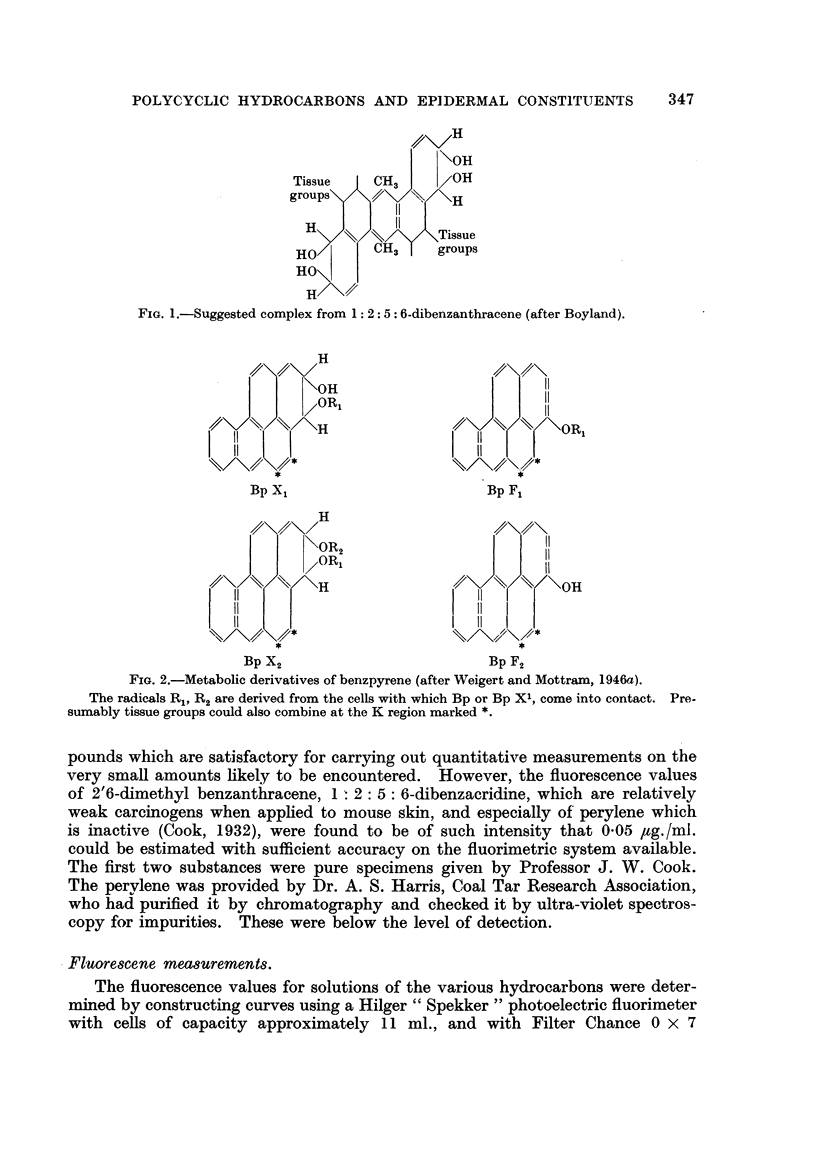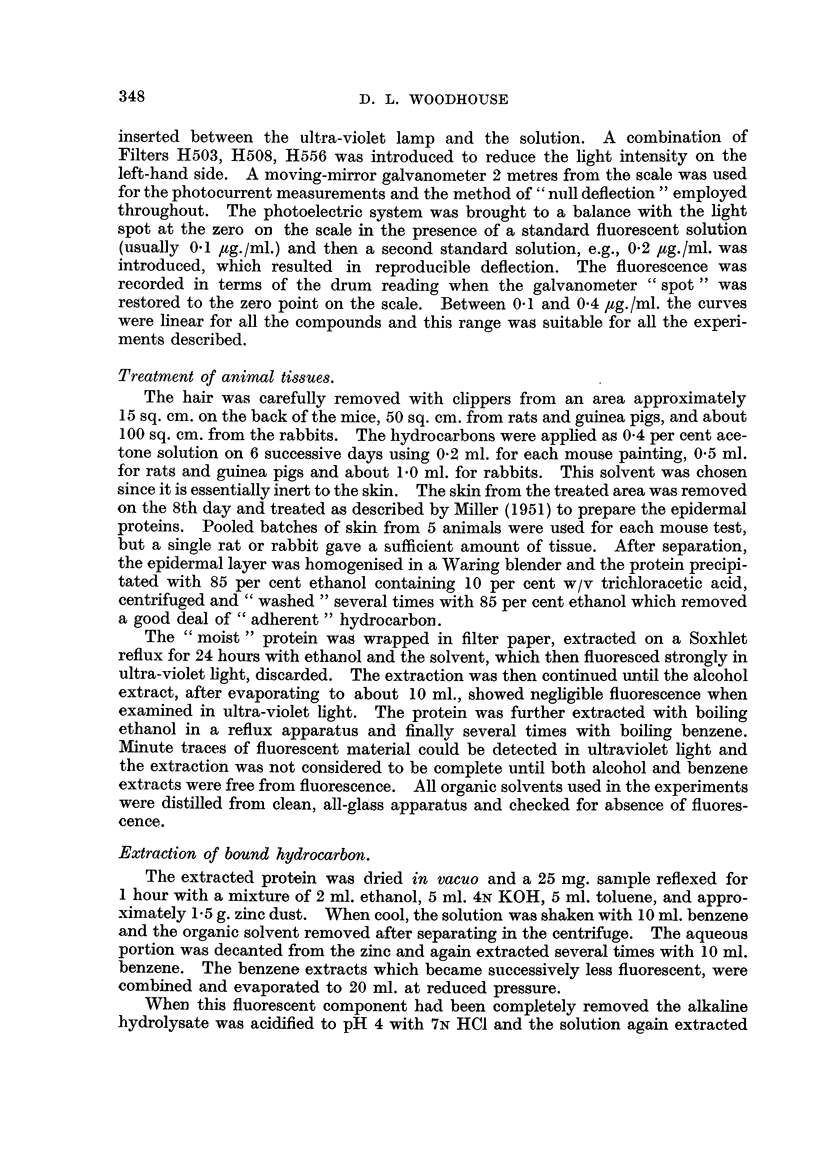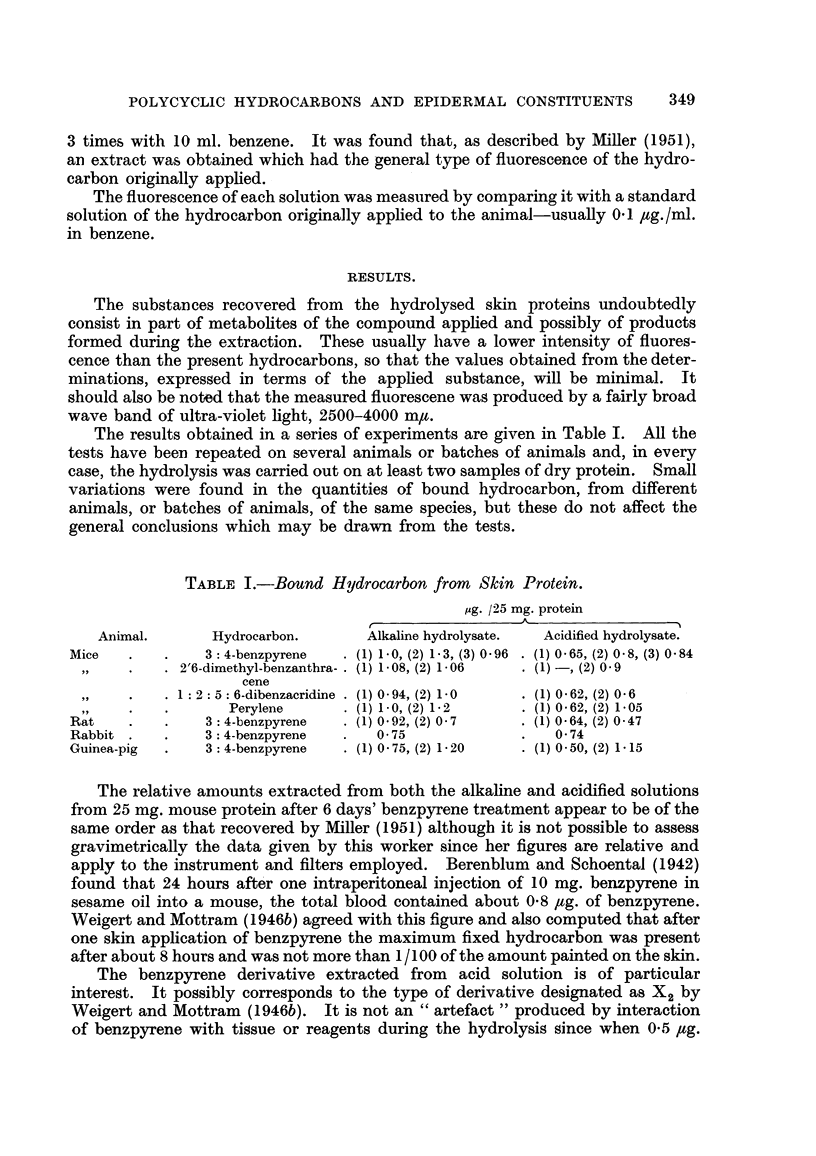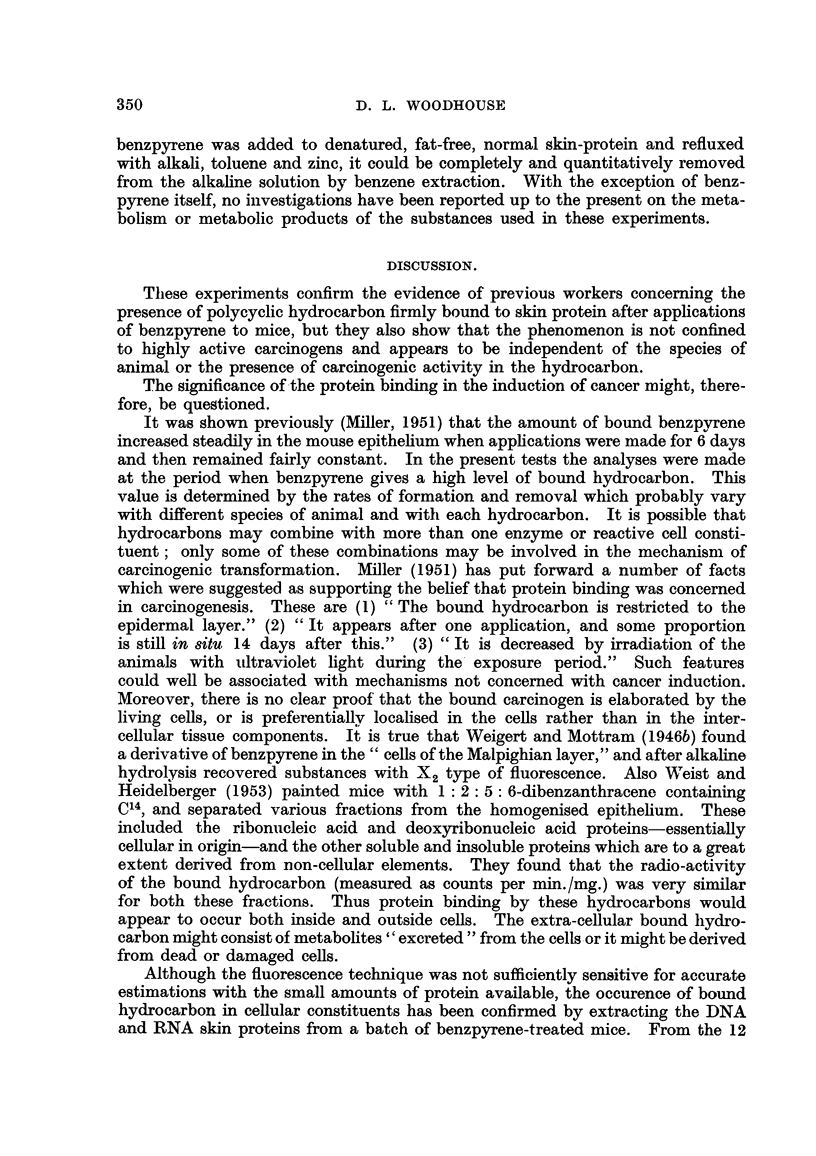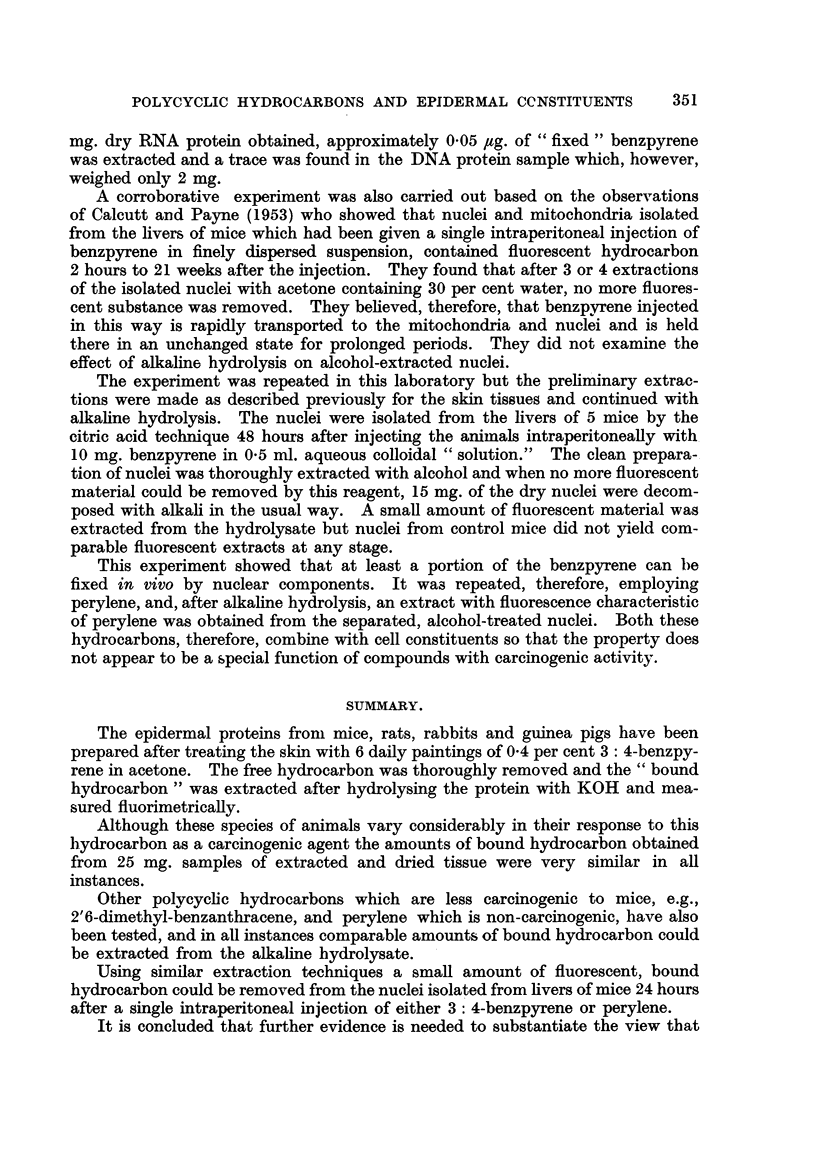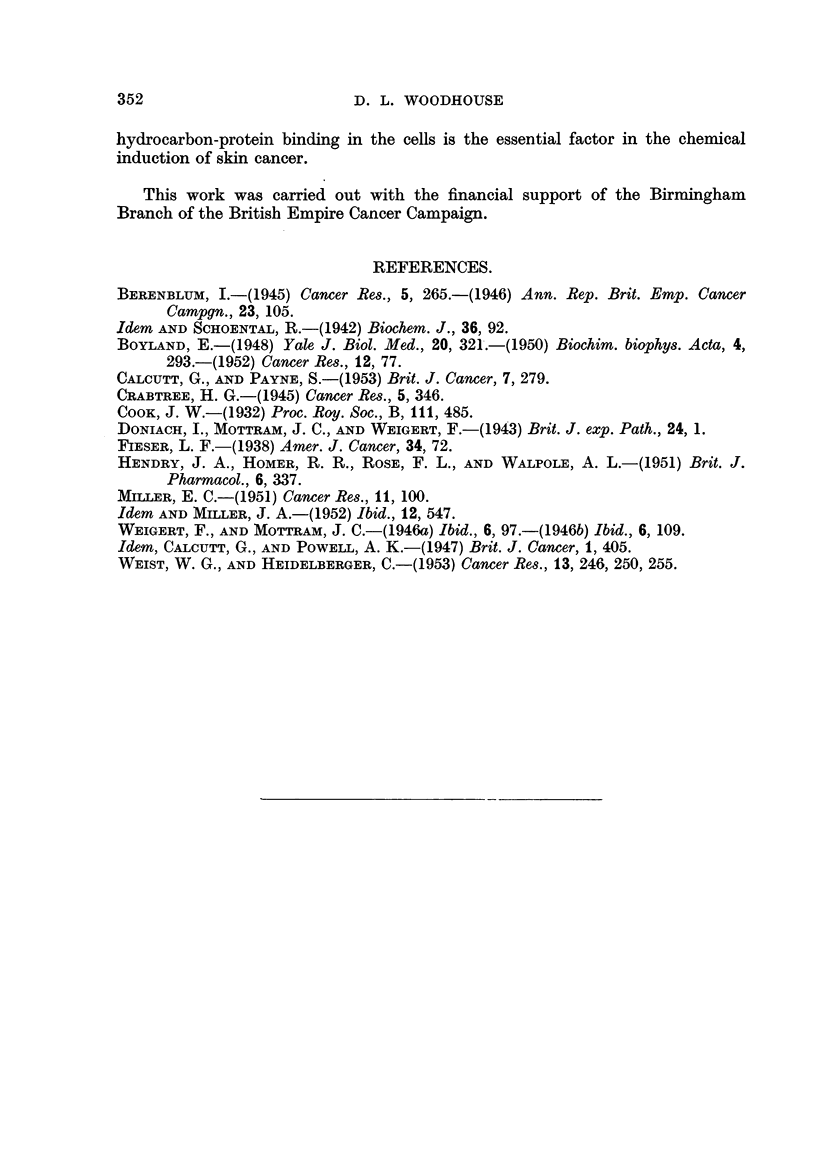# Experiments on the Interaction of Polycyclic Hydrocarbons with Epidermal Constituents

**DOI:** 10.1038/bjc.1954.35

**Published:** 1954-06

**Authors:** D. L. Woodhouse


### EXPERIMENTS ON THE INTERACTION OF POLYCYCLIC HYDROCARBONS WITH EPIDERMAL CONSTITUENTS

D. L. WOODHOUSE.

*From the Cancer Research Laboratories, Department of Pathology, The Medical School, Birmingham* 15.

Received for publication March 24, 1954.

*Experiments on the Interaction of Polycyclic Hydrocarbons with Epidermal Constituents.*

INTERPRETATIONS of the carcinogenic properties of the polycyclic hydrocarbons in terms of chemical interactions between the carcinogen and tissue constituents have been put forward by Crabtree (1945), who envisaged combination of the carcinogen with sulphur linkages as a primary phase in the process and by Boyland (1948 ; see Fig. 1 ; 1950) who suggested reaction with sulphydryl groups of enzymes necessary for metabolism of deoxypentose nucleic acid, or actual combination with nucleic acid (Boyland, 1952). Hendry, Homer, Rose and Walpole (1951) also believed that their polymerisation theory of carcinogenesis would involve combination with tissue protein. Recently, Miller and Miller (1952) have produced a good deal of evidence showing that certain polycyclic hydrocarbon carcinogens bind with skin proteins. Their results have been confirmed and extended by Weist and Heidelberger (1953) using 1 : 2 : 5 : 6-dibenzanthracene containing $C^{14}$. Valuable earlier evidence that 3 : 4-benzpyrene or its metabolites become intimately bound to skin proteins was given by the extensive researches of Weigert and Mottram who recovered four types of metabolic products from the organs of benzpyrene-injected mice, which they designated $X_1$, $X_2$, $F_1$, $F_2$, (Weigert and Mottram, 1946*a*, 1946*b* ; Doniach, Mottram, and Weigert, 1943 ; Weigert, Calcutt and Powell, 1947), the hypothetical radicals $R_1$ $R_2$ (Fig. 2) being " derived from the cells with which benzpyrene or benzpyrene $X_1$ come into contact."

The hypothesis that the production of skin cancer by such chemical agents is related to their ability to combine with tissue components at present relies almost entirely upon mouse experiments using potent carcinogens 3 : 4-benzpyrene and 1 : 2 : 5 : 6-dibenzanthracene. It seemed important, therefore, to assess the binding capacities of some polycyclic hydrocarbons allied to 1 : 2 : 5 : 6-dibenzanthracene but which are non-carcinogenic to mice, and also to determine the level of " bound " hydrocarbon, after comparable treatment, in the epidermal tissues of animals such as rats and rabbits which are less sensitive than mice to tumour induction by benzpyrene (Berenblum, 1945, 1946 ; Fieser, 1938).

### EXPERIMENTAL METHODS.

*Compounds used.*

The fluorescence of solutions of many of the inactive or less potent skin carcinogens is much weaker than that of benzpyrene. This limits the choice of com-

FIG. 1.—Suggested complex from 1 : 2 : 5 : 6-dibenzanthracene (after Boyland).

FIG. 2.—Metabolic derivatives of benzpyrene (after Weigert and Mottram, 1946*a*).

The radicals $R_1$, $R_2$ are derived from the cells with which Bp or Bp $X^1$, come into contact. Presumably tissue groups could also combine at the K region marked *.

pounds which are satisfactory for carrying out quantitative measurements on the very small amounts likely to be encountered. However, the fluorescence values of 2′6-dimethyl benzanthracene, 1 : 2 : 5 : 6-dibenzacridine, which are relatively weak carcinogens when applied to mouse skin, and especially of perylene which is inactive (Cook, 1932), were found to be of such intensity that 0·05 μg./ml. could be estimated with sufficient accuracy on the fluorimetric system available. The first two substances were pure specimens given by Professor J. W. Cook. The perylene was provided by Dr. A. S. Harris, Coal Tar Research Association, who had purified it by chromatography and checked it by ultra-violet spectroscopy for impurities. These were below the level of detection.

*Fluorescene measurements.*

The fluorescence values for solutions of the various hydrocarbons were determined by constructing curves using a Hilger " Spekker " photoelectric fluorimeter with cells of capacity approximately 11 ml., and with Filter Chance 0 × 7

inserted between the ultra-violet lamp and the solution. A combination of Filters H503, H508, H556 was introduced to reduce the light intensity on the left-hand side. A moving-mirror galvanometer 2 metres from the scale was used for the photocurrent measurements and the method of "null deflection" employed throughout. The photoelectric system was brought to a balance with the light spot at the zero on the scale in the presence of a standard fluorescent solution (usually 0·1 $\mu$g./ml.) and then a second standard solution, e.g., 0·2 $\mu$g./ml. was introduced, which resulted in reproducible deflection. The fluorescence was recorded in terms of the drum reading when the galvanometer "spot" was restored to the zero point on the scale. Between 0·1 and 0·4 $\mu$g./ml. the curves were linear for all the compounds and this range was suitable for all the experiments described.

*Treatment of animal tissues.*

The hair was carefully removed with clippers from an area approximately 15 sq. cm. on the back of the mice, 50 sq. cm. from rats and guinea pigs, and about 100 sq. cm. from the rabbits. The hydrocarbons were applied as 0·4 per cent acetone solution on 6 successive days using 0·2 ml. for each mouse painting, 0·5 ml. for rats and guinea pigs and about 1·0 ml. for rabbits. This solvent was chosen since it is essentially inert to the skin. The skin from the treated area was removed on the 8th day and treated as described by Miller (1951) to prepare the epidermal proteins. Pooled batches of skin from 5 animals were used for each mouse test, but a single rat or rabbit gave a sufficient amount of tissue. After separation, the epidermal layer was homogenised in a Waring blender and the protein precipitated with 85 per cent ethanol containing 10 per cent w/v trichloracetic acid, centrifuged and "washed" several times with 85 per cent ethanol which removed a good deal of "adherent" hydrocarbon.

The "moist" protein was wrapped in filter paper, extracted on a Soxhlet reflux for 24 hours with ethanol and the solvent, which then fluoresced strongly in ultra-violet light, discarded. The extraction was then continued until the alcohol extract, after evaporating to about 10 ml., showed negligible fluorescence when examined in ultra-violet light. The protein was further extracted with boiling ethanol in a reflux apparatus and finally several times with boiling benzene. Minute traces of fluorescent material could be detected in ultraviolet light and the extraction was not considered to be complete until both alcohol and benzene extracts were free from fluorescence. All organic solvents used in the experiments were distilled from clean, all-glass apparatus and checked for absence of fluorescence.

*Extraction of bound hydrocarbon.*

The extracted protein was dried *in vacuo* and a 25 mg. sample reflexed for 1 hour with a mixture of 2 ml. ethanol, 5 ml. 4N KOH, 5 ml. toluene, and approximately 1·5 g. zinc dust. When cool, the solution was shaken with 10 ml. benzene and the organic solvent removed after separating in the centrifuge. The aqueous portion was decanted from the zinc and again extracted several times with 10 ml. benzene. The benzene extracts which became successively less fluorescent, were combined and evaporated to 20 ml. at reduced pressure.

When this fluorescent component had been completely removed the alkaline hydrolysate was acidified to pH 4 with 7N HCl and the solution again extracted

3 times with 10 ml. benzene. It was found that, as described by Miller (1951), an extract was obtained which had the general type of fluorescence of the hydrocarbon originally applied.

The fluorescence of each solution was measured by comparing it with a standard solution of the hydrocarbon originally applied to the animal—usually 0·1 μg./ml. in benzene.

### RESULTS.

The substances recovered from the hydrolysed skin proteins undoubtedly consist in part of metabolites of the compound applied and possibly of products formed during the extraction. These usually have a lower intensity of fluorescence than the present hydrocarbons, so that the values obtained from the determinations, expressed in terms of the applied substance, will be minimal. It should also be noted that the measured fluorescene was produced by a fairly broad wave band of ultra-violet light, 2500–4000 mμ.

The results obtained in a series of experiments are given in Table I. All the tests have been repeated on several animals or batches of animals and, in every case, the hydrolysis was carried out on at least two samples of dry protein. Small variations were found in the quantities of bound hydrocarbon, from different animals, or batches of animals, of the same species, but these do not affect the general conclusions which may be drawn from the tests.

TABLE I.—*Bound Hydrocarbon from Skin Protein.*

| | | μg. /25 mg. protein | |
|---|---|---|---|
| Animal. | Hydrocarbon. | Alkaline hydrolysate. | Acidified hydrolysate. |
| Mice | 3 : 4-benzpyrene | (1) 1·0, (2) 1·3, (3) 0·96 | (1) 0·65, (2) 0·8, (3) 0·84 |
| ,, | 2′6-dimethyl-benzanthracene | (1) 1·08, (2) 1·06 | (1) —, (2) 0·9 |
| ,, | 1 : 2 : 5 : 6-dibenzacridine | (1) 0·94, (2) 1·0 | (1) 0·62, (2) 0·6 |
| ,, | Perylene | (1) 1·0, (2) 1·2 | (1) 0·62, (2) 1·05 |
| Rat | 3 : 4-benzpyrene | (1) 0·92, (2) 0·7 | (1) 0·64, (2) 0·47 |
| Rabbit | 3 : 4-benzpyrene | 0·75 | 0·74 |
| Guinea-pig | 3 : 4-benzpyrene | (1) 0·75, (2) 1·20 | (1) 0·50, (2) 1·15 |

The relative amounts extracted from both the alkaline and acidified solutions from 25 mg. mouse protein after 6 days' benzpyrene treatment appear to be of the same order as that recovered by Miller (1951) although it is not possible to assess gravimetrically the data given by this worker since her figures are relative and apply to the instrument and filters employed. Berenblum and Schoental (1942) found that 24 hours after one intraperitoneal injection of 10 mg. benzpyrene in sesame oil into a mouse, the total blood contained about 0·8 μg. of benzpyrene. Weigert and Mottram (1946*b*) agreed with this figure and also computed that after one skin application of benzpyrene the maximum fixed hydrocarbon was present after about 8 hours and was not more than 1/100 of the amount painted on the skin.

The benzpyrene derivative extracted from acid solution is of particular interest. It possibly corresponds to the type of derivative designated as $X_2$ by Weigert and Mottram (1946*b*). It is not an " artefact " produced by interaction of benzpyrene with tissue or reagents during the hydrolysis since when 0·5 μg.

benzpyrene was added to denatured, fat-free, normal skin-protein and refluxed with alkali, toluene and zinc, it could be completely and quantitatively removed from the alkaline solution by benzene extraction. With the exception of benzpyrene itself, no investigations have been reported up to the present on the metabolism or metabolic products of the substances used in these experiments.

### DISCUSSION.

These experiments confirm the evidence of previous workers concerning the presence of polycyclic hydrocarbon firmly bound to skin protein after applications of benzpyrene to mice, but they also show that the phenomenon is not confined to highly active carcinogens and appears to be independent of the species of animal or the presence of carcinogenic activity in the hydrocarbon.

The significance of the protein binding in the induction of cancer might, therefore, be questioned.

It was shown previously (Miller, 1951) that the amount of bound benzpyrene increased steadily in the mouse epithelium when applications were made for 6 days and then remained fairly constant. In the present tests the analyses were made at the period when benzpyrene gives a high level of bound hydrocarbon. This value is determined by the rates of formation and removal which probably vary with different species of animal and with each hydrocarbon. It is possible that hydrocarbons may combine with more than one enzyme or reactive cell constituent ; only some of these combinations may be involved in the mechanism of carcinogenic transformation. Miller (1951) has put forward a number of facts which were suggested as supporting the belief that protein binding was concerned in carcinogenesis. These are (1) " The bound hydrocarbon is restricted to the epidermal layer." (2) " It appears after one application, and some proportion is still *in situ* 14 days after this." (3) " It is decreased by irradiation of the animals with ultraviolet light during the exposure period." Such features could well be associated with mechanisms not concerned with cancer induction. Moreover, there is no clear proof that the bound carcinogen is elaborated by the living cells, or is preferentially localised in the cells rather than in the intercellular tissue components. It is true that Weigert and Mottram (1946*b*) found a derivative of benzpyrene in the " cells of the Malpighian layer," and after alkaline hydrolysis recovered substances with $X_2$ type of fluorescence. Also Weist and Heidelberger (1953) painted mice with 1 : 2 : 5 : 6-dibenzanthracene containing $C^{14}$, and separated various fractions from the homogenised epithelium. These included the ribonucleic acid and deoxyribonucleic acid proteins—essentially cellular in origin—and the other soluble and insoluble proteins which are to a great extent derived from non-cellular elements. They found that the radio-activity of the bound hydrocarbon (measured as counts per min./mg.) was very similar for both these fractions. Thus protein binding by these hydrocarbons would appear to occur both inside and outside cells. The extra-cellular bound hydrocarbon might consist of metabolites " excreted " from the cells or it might be derived from dead or damaged cells.

Although the fluorescence technique was not sufficiently sensitive for accurate estimations with the small amounts of protein available, the occurence of bound hydrocarbon in cellular constituents has been confirmed by extracting the DNA and RNA skin proteins from a batch of benzpyrene-treated mice. From the 12

mg. dry RNA protein obtained, approximately 0·05 $\mu$g. of " fixed " benzpyrene was extracted and a trace was found in the DNA protein sample which, however, weighed only 2 mg.

A corroborative experiment was also carried out based on the observations of Calcutt and Payne (1953) who showed that nuclei and mitochondria isolated from the livers of mice which had been given a single intraperitoneal injection of benzpyrene in finely dispersed suspension, contained fluorescent hydrocarbon 2 hours to 21 weeks after the injection. They found that after 3 or 4 extractions of the isolated nuclei with acetone containing 30 per cent water, no more fluorescent substance was removed. They believed, therefore, that benzpyrene injected in this way is rapidly transported to the mitochondria and nuclei and is held there in an unchanged state for prolonged periods. They did not examine the effect of alkaline hydrolysis on alcohol-extracted nuclei.

The experiment was repeated in this laboratory but the preliminary extractions were made as described previously for the skin tissues and continued with alkaline hydrolysis. The nuclei were isolated from the livers of 5 mice by the citric acid technique 48 hours after injecting the animals intraperitoneally with 10 mg. benzpyrene in 0·5 ml. aqueous colloidal " solution." The clean preparation of nuclei was thoroughly extracted with alcohol and when no more fluorescent material could be removed by this reagent, 15 mg. of the dry nuclei were decomposed with alkali in the usual way. A small amount of fluorescent material was extracted from the hydrolysate but nuclei from control mice did not yield comparable fluorescent extracts at any stage.

This experiment showed that at least a portion of the benzpyrene can be fixed *in vivo* by nuclear components. It was repeated, therefore, employing perylene, and, after alkaline hydrolysis, an extract with fluorescence characteristic of perylene was obtained from the separated, alcohol-treated nuclei. Both these hydrocarbons, therefore, combine with cell constituents so that the property does not appear to be a special function of compounds with carcinogenic activity.

### SUMMARY.

The epidermal proteins from mice, rats, rabbits and guinea pigs have been prepared after treating the skin with 6 daily paintings of 0·4 per cent 3 : 4-benzpyrene in acetone. The free hydrocarbon was thoroughly removed and the " bound hydrocarbon " was extracted after hydrolysing the protein with KOH and measured fluorimetrically.

Although these species of animals vary considerably in their response to this hydrocarbon as a carcinogenic agent the amounts of bound hydrocarbon obtained from 25 mg. samples of extracted and dried tissue were very similar in all instances.

Other polycyclic hydrocarbons which are less carcinogenic to mice, e.g., 2′6-dimethyl-benzanthracene, and perylene which is non-carcinogenic, have also been tested, and in all instances comparable amounts of bound hydrocarbon could be extracted from the alkaline hydrolysate.

Using similar extraction techniques a small amount of fluorescent, bound hydrocarbon could be removed from the nuclei isolated from livers of mice 24 hours after a single intraperitoneal injection of either 3 : 4-benzpyrene or perylene.

It is concluded that further evidence is needed to substantiate the view that hydrocarbon-protein binding in the cells is the essential factor in the chemical induction of skin cancer.

This work was carried out with the financial support of the Birmingham Branch of the British Empire Cancer Campaign.

### REFERENCES.

BERENBLUM, I.—(1945) *Cancer Res.*, **5**, 265.—(1946) *Ann. Rep. Brit. Emp. Cancer Campgn.*, **23**, 105.

*Idem* AND SCHOENTAL, R.—(1942) *Biochem. J.*, **36**, 92.

BOYLAND, E.—(1948) *Yale J. Biol. Med.*, **20**, 321.—(1950) *Biochim. biophys. Acta*, **4**, 293.—(1952) *Cancer Res.*, **12**, 77.

CALCUTT, G., AND PAYNE, S.—(1953) *Brit. J. Cancer*, **7**, 279.

CRABTREE, H. G.—(1945) *Cancer Res.*, **5**, 346.

COOK, J. W.—(1932) *Proc. Roy. Soc.*, B, **111**, 485.

DONIACH, I., MOTTRAM, J. C., AND WEIGERT, F.—(1943) *Brit. J. exp. Path.*, **24**, 1.

FIESER, L. F.—(1938) *Amer. J. Cancer*, **34**, 72.

HENDRY, J. A., HOMER, R. R., ROSE, F. L., AND WALPOLE, A. L.—(1951) *Brit. J. Pharmacol.*, **6**, 337.

MILLER, E. C.—(1951) *Cancer Res.*, **11**, 100.

*Idem* AND MILLER, J. A.—(1952) *Ibid.*, **12**, 547.

WEIGERT, F., AND MOTTRAM, J. C.—(1946*a*) *Ibid.*, **6**, 97.—(1946*b*) *Ibid.*, **6**, 109.

*Idem*, CALCUTT, G., AND POWELL, A. K.—(1947) *Brit. J. Cancer*, **1**, 405.

WEIST, W. G., AND HEIDELBERGER, C.—(1953) *Cancer Res.*, **13**, 246, 250, 255.